# Comparing the responses of grain fed feedlot cattle under moderate heat load and during subsequent recovery with those of feed restricted thermoneutral counterparts: metabolic hormones

**DOI:** 10.1007/s00484-023-02464-w

**Published:** 2023-04-11

**Authors:** G. Wijffels, M. L. Sullivan, S. Stockwell, S. Briscoe, S. T. Anderson, Y. Li, C. C. de Melo Costa, R. McCulloch, J. C. W. Olm, J. Cawdell-Smith, J. B. Gaughan

**Affiliations:** 1 CSIRO Agriculture and Food, Queensland Bioscience Precinct, St Lucia, Qld 4067 Australia; 2 School of Agriculture and Food, The University of Queensland, Gatton, Qld 4343 Australia; 3 School of Biomedical Sciences, The University of Queensland, St Lucia, Qld 4067 Australia; 4 Faculty of Agricultural and Veterinary Sciences, Universidade Estadual Paulista, Jaboticabal, São Paulo, Brazil; 5 School of Veterinary Science, The University of Queensland, Gatton, Qld 4343 Australia

**Keywords:** Adiponectin, Cattle, Hyperthermia, Hormones, Insulin, Leptin, Prolactin

## Abstract

We set out to determine the impact of moderate heat load on the plasma concentrations of a suite of hormones involved in regulating energy metabolism and feed intake. The responses of the thermally challenged (TC) feedlot steers were compared to those of feed restricted thermoneutral (FRTN) steers. Two sequential cohorts of twelve 518 ± 23 kg Black Angus steers on finisher grain ration were housed in climate-controlled rooms (CCR) for 18 days and returned to outdoor pens for 40 days. The TC group was subjected to a diurnal range of 28–35 °C for 7 days (Challenge) but held in thermoneutral conditions beforehand (PreChallenge), and in Recovery (after Challenge). The FRTN group was held in thermoneutral conditions and feed restricted throughout. Blood was collected over the three periods in CCR and two periods in outdoor pens for 40 days (PENS and Late PENS). Plasma concentrations of prolactin, thyroid stimulating hormone, insulin, leptin, adiponectin and thyroxine (T4) were determined during the five periods. Whilst the pituitary hormones were relatively stable, there were differences in plasma leptin, adiponectin and T4 between the two groups during Challenge and Recovery, and occasionally in PENS. The interaction of the plasma hormone concentrations and rumen temperature and DMI were also investigated. Whilst the positive relationship between DMI and leptin was confirmed, we found a strong negative relationship between adiponectin and rumen temperature, and a strong positive relationship between adiponectin and dry matter intake (DMI) in the TC steers only.

**Supplementary Information:**

The online version contains supplementary material available at 10.1007/s00484-023-02464-w.

## Introduction

As global food production adapts to a warming climate, there is great imperative to understand the impacts of increased heat load in high value production animals. Decades of research on the impacts of heat stress have documented its detrimental consequences on animal health and production (Silanikove 2000; Collier et al. 2006; 2017; Mayorga et al. 2019). Reduced feed intake, increased water loss via respiration and sweating, and increased water intake are direct responses to increased heat load (reviewed Blackshaw and Blackshaw 1994; Lees et al. 2019). Return to full feed intake, production and growth can be protracted after acute heat stress (Beatty et al. 2006; Sullivan et al. 2022).

The responses to heat stress in ruminants are influenced by many animal, environmental and management factors. Moreover, earlier experiments in climate controlled facilities were hampered by the inability to control humidity and deliver diurnal temperature cycles. A major confounder in understanding the responses to increased heat load is the voluntary reduction of feed intake, as an immediate response to increased core temperature. Furthermore, there is a paucity of studies in the growing beef animal (Collier et al. 2009). Given these knowledge gaps, we conducted a series of experiments in climate-controlled rooms with grain fed Black Angus steers to investigate a wide range of responses in feedlot cattle exposed to and recovering from the impost of increased heat load. In this paper, we focus on metabolic hormones associated with energy metabolism and feed intake during and after 7-day exposure to diurnally cycled moderate heat load. The hormones in question were insulin, prolactin, adiponectin and leptin, as well as TSH and thyroxine.

Firstly, we tested the hypothesis that the hormone trajectories of moderately heat stressed feedlot steers will differ from those obtained from feed restricted thermoneutral counterparts (FRTN). We assayed the plasma hormone concentrations of Black Angus steers subjected to moderate heat load as well as those of the FRTN group. Both treatments were followed through recovery in thermoneutral conditions and then in outdoor feedlot pens for a further 40 days. In the context of Australian summer feedlot animals, moderate heat loads occur with daily maximum temperatures in the mid-30 °C range (daily maximum THI in the mid-80 s range; Danger category). Our interest was on the overall trajectories of the treatment groups through challenge, recovery and feedlot finishing, rather than acute homeostatic responses to heat stress. Thus, our secondary hypothesis was that the plasma hormone trajectories of both treatment groups will show homeorhetic behaviours through challenge (be it heat load or feed restriction) and recovery. This phenomenon was observed for physiological measures of moderate heat stress in these same animals (Sullivan et al. 2022).

A detailed description of performance and the physiological responses of grain fed Black Angus steers to moderate heat load compared to FRTN animals during the three periods in the CCR is presented in Sullivan et al. (2022). A description of the metabolic responses across all periods, as interpreted from clinical biochemistry markers, is available also (Wijffels et al. 2022). In this report we followed the trajectories of a suite of plasma hormones in these same animals through challenge, recovery and final feedlot finishing. Furthermore, we examined the relationships between the hormone concentrations and rumen temperature or dry matter intake (DMI) and found altered relationships upon imposing heat load.

## Materials and methods

### Animal experiments

**Fig. 1:**
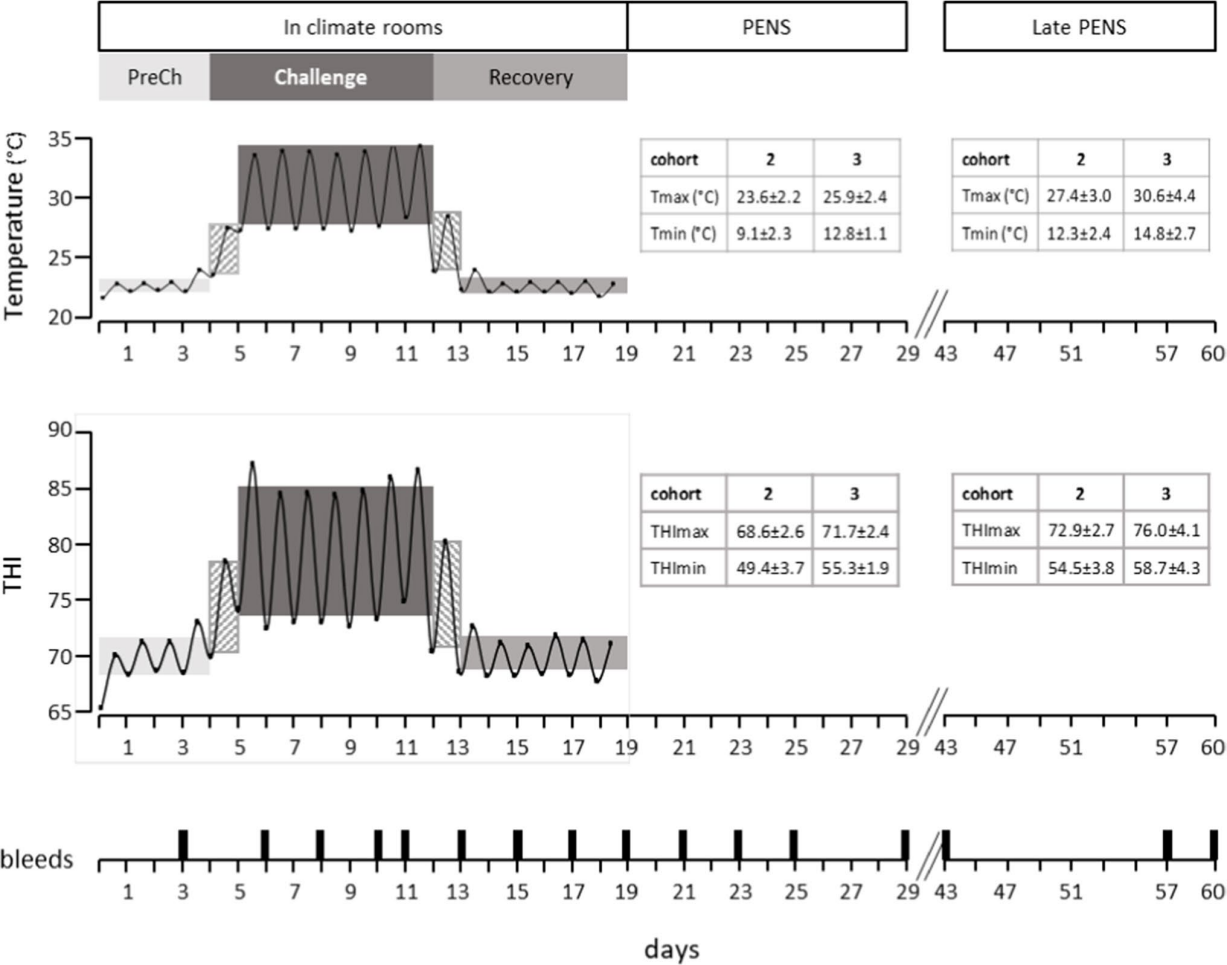
The climatic regime imposed on the Thermally Challenged (TC) group. The range of the daily ambient temperature and Temperature-Humidity Index (THI) and duration of each period is depicted for the 60 days of the experiment. The PreChallenge (PreCh), Challenge and Recovery periods were conducted in climate controlled rooms (CCR). The diurnal temperature and THI cycle experienced by the TC group is plotted for the 18 days in the CCR. The PENS and Late PENS periods occurred in outdoor feedlot pens; the climatic conditions during these periods for each of the two consecutive cohorts (cohorts 2 and 3) are presented in the inserted tables. Days of blood sampling are indicated also. Cohort 1 did not proceed through all periods, and the data from these animals was not included in the analyses for this paper (Courtesy of Wijffels et al. 2022)

In outline, two cohorts of 12 grain fed steers with live weights of 518 ± 23 kg were subjected to five sequential periods over 60 days (Fig. 1). For the first 18 days, the animals were housed in the climate controlled rooms (CCR) and allocated to two treatment groups (*n* = 6), feed restricted thermoneutral (FRTN) and thermally challenged (TC). Whilst in the CCR, they were subjected to three periods, PreChallenge, Challenge and Recovery. The FRTN group remained in thermoneutral conditions throughout the 18 days and experienced near constant air temperature (20.3 °C), percent relative humidity (%RH, 71.5%) and Temperature-Humidity Index (THI, 69). During the 4 days of PreChallenge, the TC group experienced mean air temperature and %RH of 22.7 °C and 64%, giving a mean THI of 70. A highly detailed methodology depicting the animal treatments whilst in the CCR is given by Sullivan et al. (2022), and a summarised version with information of the treatment during outdoor feedlot finishing is presented in Wijffels et al. (2022).

The 7-day Challenge period for the TC group was delivered with diurnal cycling: the respective mean daily maximum air temperature and %RH were 34.5 °C and 58%, and respective mean daily minimum air temperature and %RH were 27.9 °C and 36.4%. The respective mean daily maximum and minimum THI were 82.5 and 73.6 (Fig. 1). The diurnal cycles of air temperature and %RH imposed during Challenge were guided by an analysis of meteorological data for a locality that hosts a number of feedlots. Hourly air temperature and %RH data of the two preceding years were obtained for weather station 041,522 (Dalby airport, latitude − 27.1605. longitude 151.2633) from the Australian Bureau of Meteorology. Several moderate to severe heatwaves were identified, and the hourly data of each day of the heat waves were averaged to model diurnal air temperature and %RH cycles (unpublished data). The air temperature cycle revealed that morning air temperatures of 25–27 °C at 0700–0800 h rose rapidly to 32–34 °C by 1000 h. Over the same time interval, the %RH decreased from 56% RH to 32% RH. The afternoon air temperatures fell from the daytime plateau from approximately 1600 h to the night-time minimum which occurred at variable times. In the CCR during Challenge, these conditions were accelerated so that the morning rise and plateau of daytime maximum air temperature, and conversely the fall and daytime plateau in %RH was achieved between 0750 and 0920 h. In the afternoon, the reduction in air temperature from the daytime maximum plateau to the overnight minimum temperature occurred over 1550 – 1750 h. %RH increased over this interval returning to the overnight %RH of approximately 58%. The conditions for the third and last period in the CCR, the 6-day Recovery, were similar to the PreChallenge period with air temperature held at 22.7 °C, whilst %RH ranged over 37.6 to 58.8% (mean THI of 70.3). The steers returned to outdoor feedlot pens for the remaining 40 days where they experienced late winter – mid-spring conditions (mean minimum and maximum air temperatures (± SD) of 12.9 ± 3.1 and 27.8 ± 4.1 °C respectively; mean minimum and maximum THI (± SD): 55.4 ± 4.8 and 73.2 ± 3.9; Fig. 1).

All animals were offered a grain-based feedlot finisher diet throughout the 60-day experiment. The finisher diet provided 13.1 MJ/kg metabolizable energy, 2.9% crude fat and 15.4% crude protein and was augmented with 20 mg/kg sodium monensin (Sullivan et al. 2022). The TC group were fed ad lib, whereas the FRTN group were feed restricted during PreChallenge, Challenge and Recovery, based on the feed intake and feed offered to a live weight matched pair in the TC group. The feed *offered* regime imposed on both treatment groups in the current experiment saw each live weight match pair offered the same amount of feed on the same day. The amount of feed offered was based on the feed consumed by the TC steer the previous day and an additional 20% (by weight). Simply put, if the TC steer consumed 10 kg on day 8, both the TC and TN animals of the pairing were offered 10 kg feed on day 9. This method differs from the pair fed approach applied to many animal challenge experiments. When pair feeding is imposed in thermal challenge experiments, the TN animal is fed the exact amount as that consumed by the TC counterpart on the previous day (O’Brien et al. 2010; Wheelock et al. 2010). The rationale for devising and implementing the pair *offered* regime was to avoid large sudden reductions in feed intake in the TN animals which can cause anxiety, stress and impairment of rumen function (Schwartzkopf-Genswein et al. 2003).

### Hormone assays

Concentrations of plasma prolactin, TSH, adiponectin and leptin were determined using sandwich enzyme-linked immunosorbent assays (ELISA). Assay plates (clear 384 well, Perkin Elmer, MA) were coated overnight with the relevant capture antibody in a sodium carbonate coating buffer (0.1 M Na_2_CO_3_ pH 9.6) at 4 °C. Recombinant protein standards were serially diluted to appropriate concentrations in either a pooled clarified calf serum (leptin and TSH) or commercial horse herd serum (prolactin and adiponectin). Plasma samples and standards were then diluted further as required in Tris-buffered saline containing Tween 20 (TBST: 50 mM Tris, 150 mM NaCl, pH 7.6 and 0.1% Tween 20). EDTA treated plasma was used in all assays.

All reagents were delivered using an EpMotion 5075 liquid handling robot. The concentrations of capture and detection antibodies are given in Suppl. Table 1. Plates were manually washed 4 times in TBST between each step. The coated plates were blocked for 30 min in 2% skim milk powder in TBST. The samples (in duplicate) and standards (in quadruplicate) were added, and the plates were incubated for 1 h at room temperature (RT). Detection antibodies (in TBST) were added to all wells and incubated for 30 min (RT). An amplification step (30 min, RT) was included for the prolactin ELISA using Streptavidin/HRP (Pierce 1:20,000), and for the adiponectin and leptin ELISAs, anti-chicken/HRP (KPL; 1:4000) and anti-rabbit/HRP (KPL; 1:2000) antibodies were utilised respectively. Results were visualised using a 3,3′,5,5′-tetramethylbenzidine (TMB) solution (Biorad Core +) and the reaction stopped with 2% sulphuric acid. The colour development was measured at 450 nm on a Spectramax M3 plate reader (Bio-Strategy). The performance of each assay is given in Suppl. Table 2.

Plasma thyroxine (T4) levels were measured using a competitive binding ELISA. Clear 384 well plates were coated with a monoclonal antibody to thyroxine (Suppl. Table 1) overnight in carbonate coating buffer (4 °C). After washing, plates were blocked as described above. T4-BSA (SQX-CBS-8168, Squarix, Germany) was prepared in a 200 µM solution of 8-anilino-1-naphthalene sulfonic acid (ANSA) in BSA/TBST. Plasma samples (in triplicate) were diluted 1:10 in a 200 nM ANSA/DMSO solution in TBST. T4-HRP (SQRX-T4HRP.1, Squarix) was used as a competitor at 5 nM in TBST. Diluted samples (in triplicate) and standards (in quadruplicate) were added to the assay plates along with the T4-HRP and incubated (30–40 min, RT). Reagents were delivered by robot, and the plates washed as described above. Reactions were visualised with TMB, stopped with sulphuric acid and read at 450 nm.

Plasma insulin concentrations were determined using a radio-immunoassay kit (TKIN2, Coat-a-Count Insulin, Siemens Healthcare Diagnostics, CA) according to manufacturer’s protocol. The results are expressed as mIU/mL in reference to the WHO human insulin standard (IRP 66/304).

### Statistical analysis of plasma hormone concentrations and linear regression

Seven hormone concentration variables were analysed: adiponectin, log_2_(insulin), log_2_(insulin):glucose, leptin, log_2_(prolactin), T4 and log_10_(TSH). The statistical analyses methodology followed the same method as described by Wijffels et al. (2022), i.e. applying the PROC Mixed model with the REML estimation method within the SAS Program (version 9.4, SAS Institute Inc., Cary, NC; 2002–2012). Note, that as all animals underwent the same treatments until day 5, the PreChallenge mean and SEM for each variable were determined from the values collected on all animals on day 3. Simple linear regression was conducted in Prism 9.0 (GraphPad Software, San Diego, CA) to discover and describe relationships between the daily mean hormone concentration and the corresponding daily means of rumen temperature or daily mean DMI over the 18 days in the CCR. *p* values less than 0.05 were considered significant. A trend towards significance was noted over a *p* value range where 0.08 ≤ *p* ≥ 0.05.

## Results

### Hormone trajectories over the five periods

#### Pituitary hormones—TSH and prolactin

**Fig. 2:**
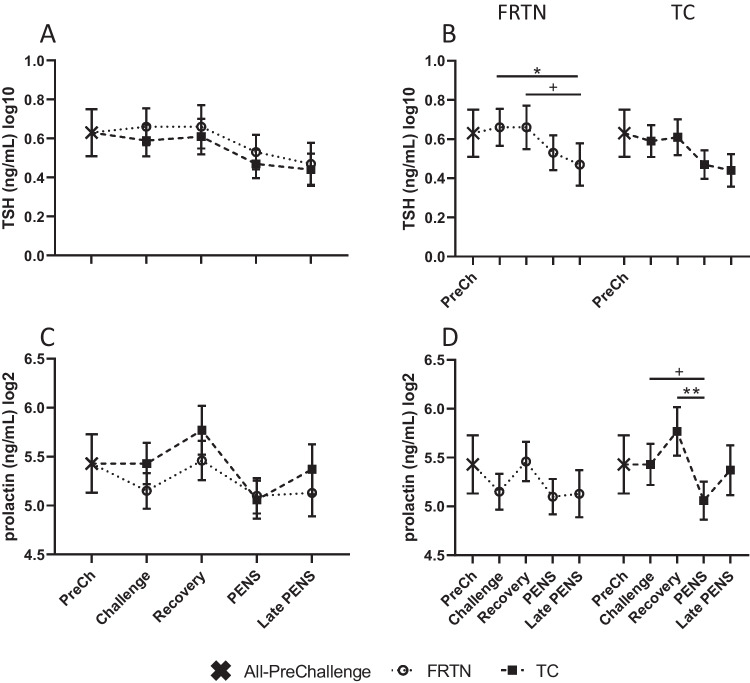
Plasma concentrations of TSH and prolactin over the five periods. The PreChallenge (PreCh) mean is denoted by X. Panels **A** and **C**. Comparison of the mean values (± SEM) of the groups for plasma TSH(log10) and prolactin(log2) concentrations respectively. Panels **B** and **D**. Within group comparisons for each group across the five periods for plasma TSH(log10) and prolactin(log2) concentrations respectively. + , *p* < 0.1; *, *p* < 0.05; **, *p* < 0.01

The daily mean plasma TSH and prolactin concentrations showed high individual animal variability and were log transformed to facilitate comparison between the two treatment groups (Fig. 2). The plasma TSH(log10) trajectories of both treatment groups were in close resemblance, and mean concentrations were not different to each other at any period (Fig. 2A, B). Similarly, for plasma prolactin whilst there was an effect of period (*p* = 0.033), there was no discernible difference between the groups at any time (Fig. 2C, D).

#### Metabolic hormones

**Fig. 3:**
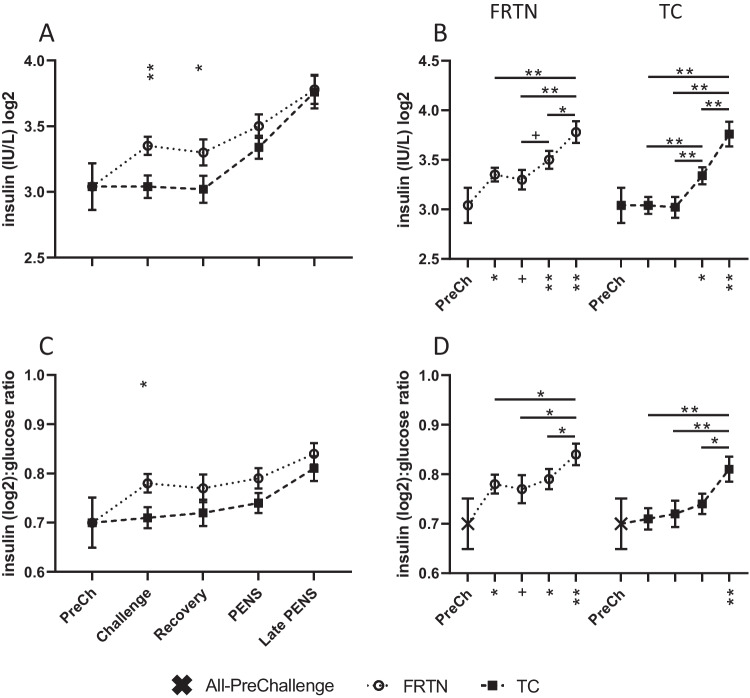
Plasma insulin concentration and the insulin: glucose ratio over the five periods. The PreChallenge (PreCh) mean is denoted by X. Panels **A** and **C**. Comparison of the mean values (± SEM) of the groups for plasma insulin(log2) concentration and the (log_2_)insulin:glucose ratio respectively. Significant differences are indicated by the asterisks above the period means. Panels **B** and **D**. Within group comparisons of plasma insulin(log2) concentrations and the (log_2_)insulin:glucose ratios respectively for each group across the five periods (PreChallenge, Challenge, Recovery, PENS and Late PENS, refer to the x-axis Fig. 2D). The asterisks under the x-axis indicate statistically significant difference with the PreChallenge mean. + , *p* < 0.1; *, *p* < 0.05; **, *p* < 0.01

Plasma insulin concentrations were highly variable in both groups during all periods. With log2 transformation, major effects of treatment (*p* = 0.009) and period (*p* < 0.0001) were identified. The mean plasma insulin concentrations in TC animals were significantly higher (~ 9%) than FRTN during Challenge and Recovery (Fig. 3A, B). In PENS and Late PENS, plasma insulin concentrations increased for both groups. The log_2_(insulin):glucose ratio also showed treatment (*p* = 0.047) and period (*p* < 0.0001) effects. The TC mean was higher than the FRTN mean during Challenge only (Fig. 3C, D). In Late PENS, the mean log_2_(insulin):glucose ratios were both increased relative to the means of all preceding periods.

**Fig. 4:**
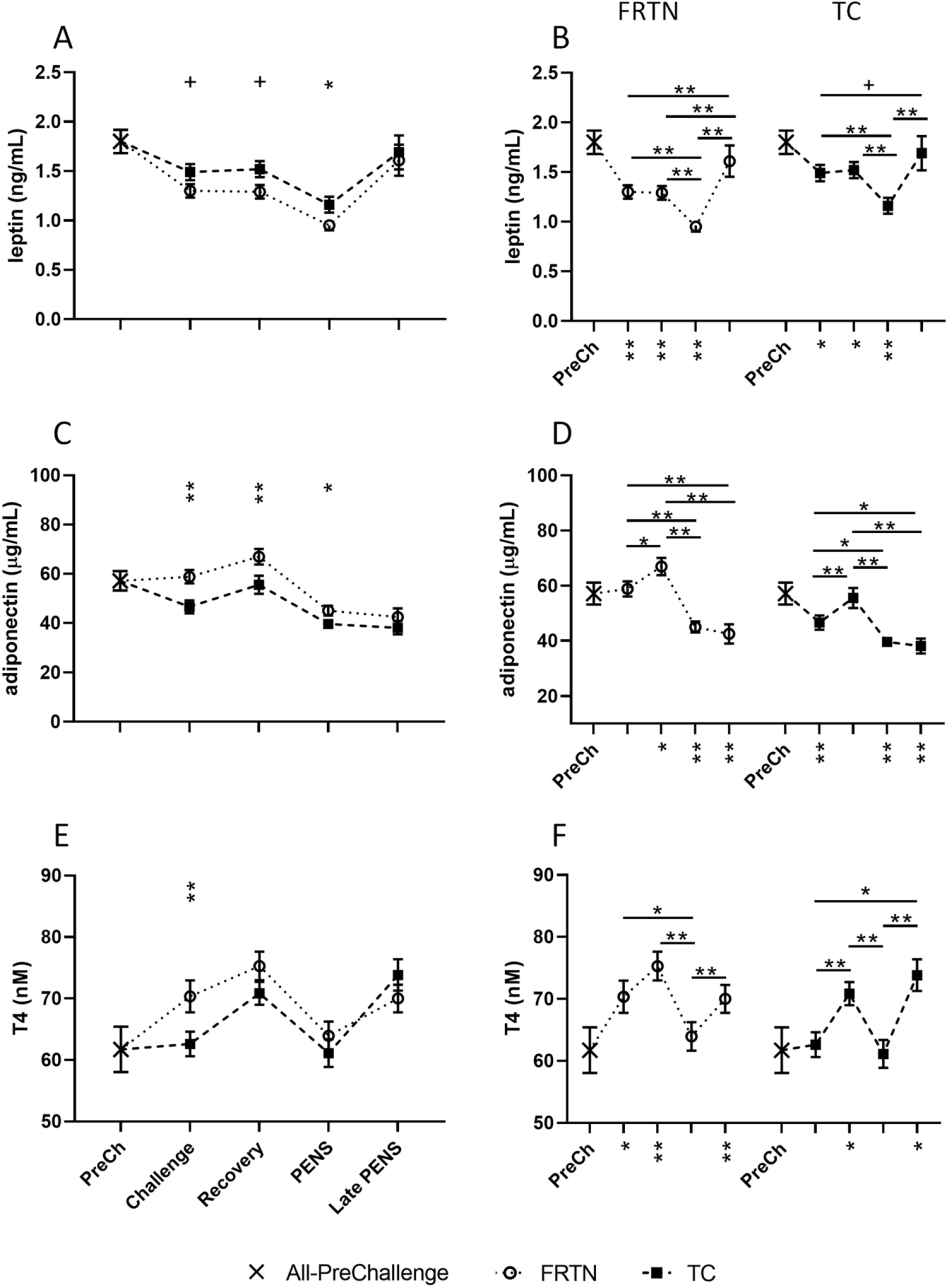
Plasma leptin, adiponectin and thyroxine (T4) concentrations over the five periods. The PreChallenge (PreCh) mean is denoted by X. Panels **A**, **C** and E. Comparison of the mean values (± SEM) of the groups for plasma leptin, adiponectin and T4 concentrations respectively. Significant differences are indicated by the asterisks above the period means. Panels **B**, **D** and F. Within group comparisons of the plasma leptin, adiponectin and T4 concentrations respectively for each group across the five periods (PreChallenge, Challenge, Recovery, PENS and Late PENS, refer to the x-axis Fig. 2D). The asterisks under the x-axis indicate statistically significant difference with the PreChallenge mean. + , *p* < 0.1; *, *p* < 0.05; **, *p* < 0.01

Changes in mean plasma leptin concentrations were comparable in both TC and FRTN groups (Fig. 4A, B). There were major effects of treatment (*p* = 0.0086) and period (*p* < 0.0001). For the TC group during Challenge through to PENS, the mean leptin concentrations were higher or tended to be higher than the FRTN means, although ~ 16–17% reduced relative to the PreChallenge mean. Whilst the leptin levels were stable in both groups in Challenge and Recovery, the mean leptin concentrations fell a further 25% for both groups in PENS. In Late PENS, leptin levels in both groups were close to the PreChallenge mean (Fig. 4A).

There were significant effects of treatment (*p* < 0.0001) and period (*p* < 0.0001) on plasma adiponectin concentration. The TC means were persistently lower than the FRTN means during Challenge, Recovery and PENS (Fig. 4C, D). Relative to the PreChallenge mean, the TC mean fell approximately 18% during Challenge. There was no change in the FRTN mean. Both groups achieved the highest adiponectin concentrations during Recovery; in PENS and Late PENS, the adiponectin concentrations stabilised at 21–27% below the PreChallenge mean.

For plasma T4 concentrations there were major effects of treatment (*p* = 0.011) and period (*p* < 0.0001). The TC group was distinctive in the absence of a rise in plasma T4 during Challenge, being 11% lower than the FRTN mean. Otherwise, the two treatment groups behaved similarly with the highest concentrations of plasma T4 occurring during Recovery (Fig. 4E, F), followed by a fall in PENS to be equivalent to the PreChallenge mean. There was a rise in Late PENS.

#### Interactions with rumen temperature and DMI during PreChallenge, Challenge and Recovery

Investigation of the relationships between the daily mean concentrations of the plasma hormones and daily mean rumen temperatures and DMI were inspired by the linear and elliptical responses discovered between rumen temperature and physiological and performance measures under moderate heat load and during recovery (Sullivan et al. 2022). During the 18 days in the CCR, the daily mean rumen temperature range for the TC group was 39.57 – 40.64 °C, whilst that of the FRTN group was 39.22 – 39.80 °C (Sullivan et al. 2022). Thus, the relationships described below can be ascribed to these ranges only.

#### Pituitary hormones—TSH and prolactin

**Fig. 5:**
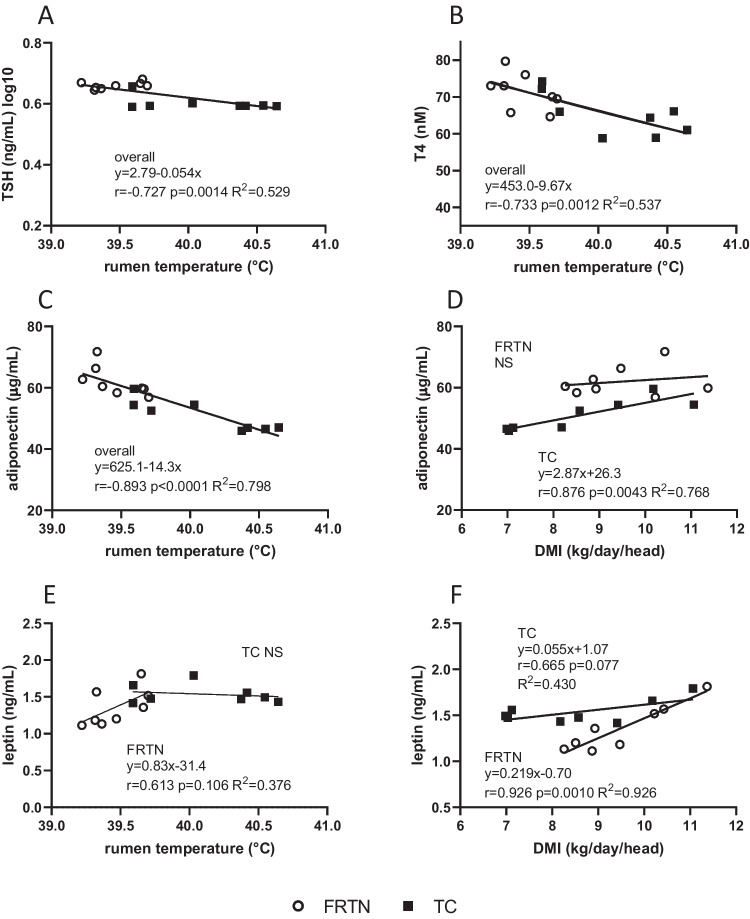
Linear relationships between daily mean plasma hormone concentrations and daily mean rumen temperature or DMI of the thermally challenged (TC) and feed restricted thermoneutral (FRTN) groups during the three periods in the CCR. **A** TSH(log10) vs rumen temperature. **B** T4 vs rumen temperature. **C** Adiponectin vs rumen temperature. **D** Adiponectin vs DMI. **E** Leptin vs rumen temperature. **F** Leptin vs DMI. The line-of-best fit and linear equation are given for the data pooled from both treatment groups (overall) or for each treatment group (TC and FRTN) along with the Pearson correlation *r*, the level of significance and the coefficient of determination, *R*^2^. NS, not significant

A negative linear relationship between daily mean rumen temperature and daily mean TSH(log10) concentrations was evident when combining the values from both treatment groups (*r* =  − 0.727, *p* = 0.0014; Fig. 5A). The highest daily mean rumen temperature (40.64 °C) was associated with a mean daily TSH concentration of 3.38 ng TSH/mL. The lowest daily mean rumen temperature (39.22 °C) occurred with a mean daily TSH concentration of 4.67 ng TSH/mL. Thus, the 1.42 °C increment in rumen temperature related to a 1.29 ng/mL fall in plasma TSH concentration, representing a fall of ~ 28% in mean daily TSH concentration between the two extremes of rumen temperature. In contrast, the daily mean prolactin(log2) concentrations did not display any relationships across the whole range of daily mean rumen temperatures obtained by the two groups (*p* = 0.929; data not shown). Neither TSH nor prolactin demonstrated a significant relationship with DMI (data not shown).

#### Metabolic hormones

The mean daily concentrations of plasma insulin(log2) and the insulin(log2):glucose ratio did not display any relationships (linear or otherwise) with daily mean rumen temperature or DMI (data not shown). The mean daily concentrations of adiponectin and T4 yielded negative linear relationships with rumen temperature across both treatment groups (Fig. 5B, C). In the case of T4, the relationship was attributed a moderate negative Pearson correlation (*r*) of − 0.733 (*p* = 0.0022), whilst that of adiponectin possessed a strong negative correlation (*r* =  − 0.985, *p* < 0.0001). Theoretically, the falls in T4 and adiponectin concentrations were at rates of 10 nM and 14 µg/mL per degree (°C) increase in daily mean rumen temperature respectively. The percent reduction in daily mean T4 concentration over the rumen temperature range was 16.4%, whilst the percent fall in daily mean adiponectin concentration was 25.0%. Due to the linear relationship between rumen temperature and water consumption in this experiment (Sullivan et al. 2022), both daily mean T4 and adiponectin concentrations displayed moderate to strong negative relationships with mean daily water consumption (Suppl. Fig. 1A and B). Daily mean leptin concentration showed a trend towards a moderately correlated positive linear relationship with rumen temperature (*p* = 0.106, *r* = 0.613) for the FRTN group only (Fig. 5E).

When assessing the interaction of metabolic hormones with DMI, relationships were discovered for adiponectin and leptin (Fig. 5D, F). No relationship was apparent for T4 concentrations (data not shown). The TC group demonstrated a positive linear relationship between mean daily adiponectin concentration and DMI (*r* = 0.876, *p* = 0.0043); for every 1 kg/day/head reduction in DMI, the daily mean adiponectin concentration fell by 2.9 µg/mL In the case of leptin, the FRTN group exhibited a strong positive relationship with DMI (*r* = 0.926, *p* = 0.0010); the rate of reduction in leptin concentration was 0.22 ng/mL for each 1 kg/day/head decrease in DMI. In contrast, the TC group showed only a moderate correlation (*r* = 0.665*,* *p* = 0.077). The rate of decrease in leptin concentration was fourfold less than that of the FRTN group at 0.055 ng/mL per kg/day/head decrement in DMI.

## Discussion

Uninterrupted feed intake and growth are critical to feedlot profitability and animal welfare. Increased heat loads are well known to reduce feed intake and weight gain (Hahn 1999; Brown-Brandl et al. 2003; Beatty et al. 2006; Sullivan et al. 2011). However, the behaviours of the hormones that influence these responses during increased heat loads are understudied especially in the feedlot animal. Moreover, most studies focus on one or two such hormones. In this study, the plasma concentrations of a suite of hormones modulating energy metabolism within tissues and at the whole animal level, as well as appetence, were followed through two differing short-term perturbations, and then through recovery and feedlot finishing in outdoor pens. The thermally challenged group (TC) during moderate heat load employed self-directed reduction in feed intake. Likewise in Recovery, the TC group self-directed re-alimentation.

### Pituitary hormones

The plasma concentrations of the two pituitary hormones, TSH and prolactin, were relatively stable across the periods regardless of treatment. Previous studies reported that circulating TSH levels were remarkably unresponsive to fasting, starvation and protein malnutrition in humans (Palmblad et al. 1977; Merimee and Fineberg 1976) and rats (Rondeel et al. 1992; van der Wal et al. 1998). The TSH concentrations we observed in FRTN animals agree with these reports. No change in TSH levels was observed with increased heat load in late gestation or early lactation dairy cows (Weitzel et al. 2017), or calves, heifers and bulls (Schams et al. 1980). At the aggregate level of periods, our data concur with these findings. Only Kahl et al. (2015) observed a 40% reduction in plasma TSH in steers after 9 days of increased heat load relative to FRTN steers. However, in the current experiment and at the level of daily means of rumen temperature and plasma TSH(log10) concentrations across all animals, a moderately correlated and negative linear relationship between the two variables was discerned. Overall, there was a substantial reduction (~ 28%) in plasma TSH concentration across the range of daily mean rumen temperatures experienced by these steers.

Prolactin has been associated with increased heat load in ruminants (reviewed by Alamer 2011) and in humans (Robins et al. 1987; Burk et al. 2012; Wright et al. 2012). Good correspondence between plasma prolactin concentrations and core temperatures (or ambient temperatures) has been shown in studies of ruminants undergoing thermal challenge (Wettemann and Tucker 1976; Sergent et al. 1985; Smith et al. 1977; Schillo et al. 1978; Scharf et al. 2010; Ronchi et al. 2001;). Moreover, some studies have indicated a role for prolactin in regulating water consumption in heat stressed ruminants (reviewed Alamer 2011). Contrarily, fasting or underfeeding can depress plasma prolactin concentrations in steers, humans and rats (Becker et al. 1985; Tegelman et al. 1986; Bergendahl et al. 1989). Ronchi et al. (2001) could not report such a response in feed restricted heifers. Clearly, the perturbations of moderate heat load and/or reduced feed intake imposed on the beef steers in the current study were not sufficient to induce an overt response in secretion of prolactin. Furthermore, no relationships were discovered for daily mean prolactin concentration with the daily means of rumen temperature, DMI and water consumption.

### Metabolic hormones

Thyroid hormones, T4 and T3, have strong influences on metabolic rate and thus endogenous heat production. Five-day fasted sheep and cattle experienced reduced plasma T3 and T4 concentrations (Blum et al. 1980; Blum and Kunz 1981). Unusually, the FRTN group during Challenge (reduced feed intake) and Recovery (realimentation) experienced a rise in plasma T4, which then fell to PreChallenge levels in PENS. The increased plasma T4 might possibly be a manifestation of a confinement stress, i.e. being held in climate rooms for a prolonged period combined with reduced feed on offer. This argument is supported by a small transient rise in rumen temperature detected over days 3–6 in the FRTN group, indicative of a stress induced hyperthermia (Sullivan et al. 2022). Confinement of lambs and calves induced sharp rises in plasma thyroid hormone concentrations (Friend et al. 1985; 1987; Bowers et al. 1993) as does sudden change in animal handling practices (Pierzchala et al. 1983; Falconer 1976). According to Chatzitomaris et al. (2017) this is a classic type 2 allostatic load response by healthy animals anticipating increased energy expenditure. So, despite an average 25% decrease in DMI imposed on the FRTN group, the allostatic load response dominated. When returned to outdoor pens, with the confinement/handling stress removed, plasma T4 levels fell to PreChallenge levels.

The TC group exhibited an apparent absence of a T4 response during Challenge. There was no equivalent rise in plasma T4, and the rise in Recovery was muted relative to the FRTN group. Typically, increased heat load depresses thyroid hormone secretion (Johnson and Ragsdale 1960; Valtorta et al. 1982; Magdub et al. 1982; Baccari et al. 1983; O’Kelly 1986; Pereira et al. 2008; Kahl et al. 2015; Weitzel et al. 2017). When comparison was made with pair-fed thermoneutral (PFTN) animals to their heat stressed counterparts, the reduction in plasma thyroid concentrations was greater in the latter treatment group (Valtorta et al. 1982; Kahl et al. 2015; Weitzel et al. 2017). The lack of plasma T4 response in the TC group during heat load in our study might be interpreted as a consequence of a depression in thyroid output due to increased heat load counteracted by an elevation induced by the type 2 allostatic response. Once the heat load was removed, the T4 trajectory of the TC group paralleled that of the FRTN group. The interaction of plasma T4 levels and core temperature was corroborated by a linear relationship with daily mean rumen temperature across the rumen temperature range experienced by the steers. In Late PENS, plasma T4 levels rose again in both treatment groups. Increased thyroid activity and T4 levels above basal levels are frequently observed during late compensatory gain (Blum et al. 1980; 1985; Cabaraux et al. 2003; Valtorta et al. 1982; Baccari et al. 1983). This seems to be the case for both treatment groups in this study.

The insulin response of the FRTN group also suggests a confinement and/or handling stress. Plasma insulin during Challenge and Recovery showed a small but significant increase, along with an increased insulin:glucose ratio during Challenge. A *reduction* in plasma insulin is anticipated as a consequence of reduced feed intake. Short-term fasted steers and lambs experience substantial reductions in basal insulin levels which recover quickly on refeeding (Blum and Kunz 1981; Rule et al. 1985; Cole et al. 1988). PFTN lactating cows when underfed produced either no change or a fall in plasma insulin (Baumgard et al. 2011; Wheelock et al. 2010). However, acute stress induces elevated plasma insulin levels in rodent models (Rostamkhani et al. 2012; Depke et al. 2008; Ricart-Jané et al. 2002).

The TC group experienced no change in plasma insulin during Challenge and Recovery. So, if the rise in plasma insulin in the FRTN group reflected a confinement stress and reduced feed on offer, then it is clear that the TC animals did not perceive or respond to that stress via insulin secretion. Insulin responses to heat stress in ruminants are inconsistent revealing the many factors involved in regulating its secretion. For the heat stressed lactating dairy cow all manner of responses to various thermal challenges have been reported (Hall et al. 2018; Garner et al. 2017; Itoh et al. 1998a; Wheelock et al. 2010; Baumgard et al. 2011; Koch et al. 2016). Reduced plasma insulin as a consequence of heat stress has been observed in the heat stressed non-lactating cow (Itoh et al. 1998b; Koch et al. 2016), but no response was recorded for heifers (Itoh et al. 1998c). Thermally challenged beef calves tended to higher plasma insulin relative to PFTN counterparts but then there was an increase in plasma insulin in both groups (O’Brien et al. 2010). In our study, as DMI and live weight rose in PENS and Late PENS, plasma insulin levels for both treatment groups increased in a comparable manner. The upward trajectories of the plasma insulin concentrations and the (log_2_)insulin: glucose ratios over these periods are likely to indicate insulin resistance not uncommon in finishing feedlot cattle (Kneeskern et al. 2016; reviewed DiGiacomo et al. 2014).

Leptin is an anorexigenic protein hormone mostly secreted from white adipose tissue into circulation, and as such plays a role in signalling to the hypothalamus of the availability of energy reserves stored as fat. It is therefore implicated in the regulation of feed intake, energy metabolism and readiness for reproduction (reviewed Chilliard et al. 2005; D’souza et al. 2017). In both groups, plasma leptin concentrations were reduced during Challenge, Recovery and PENS, but the TC group experienced a weaker response. The reduced plasma leptin in the FRTN group during Challenge (i.e. feed restriction) and Recovery (realimentation) is typical of feed restricted or underfed ruminants (Chilliard et al. 2005).

The reduction in plasma leptin by the TC group during Challenge and Recovery was only half that of the FRTN mean. Mean DMI for the TC group was 2 kg/head/day less than the FRTN group over those same two periods (*p* < 0.0001). Despite the lower feed intake, the TC group exhibited higher plasma leptin concentrations which may contribute to the low appetence during increased thermal load. A rise in plasma leptin is more frequently reported in heat stressed ruminants (Scharf et al. 2010; Al-Dawood 2017; Bernabucci et al. 2011) although Garner et al. (2017) found no change to plasma leptin levels in short-term heat stressed lactating cows. In the current study, the relationship between plasma leptin and rumen temperature suggested a distinct threshold at approximately 39.7 °C, after which leptin concentration was stable; however, the threshold was only evident due to the fall in daily rumen temperature in the FRTN group as DMI fell (Sullivan et al. 2022).

Plasma leptin concentration has been shown to have a strong positive relationship with DMI in the context of the lot fed beef steer (Foote et al. 2016). We found that this relationship was altered by heat load; the reduction of DMI by the TC group was associated with a corresponding rate of fall in plasma leptin concentration that was fourfold less than that of the FRTN group. The restrained lowering of plasma leptin in the TC group would have influenced appetence. In an animal trying to reduce endogenous heat production, lessening the rumen fermentative and metabolic heat loads by downregulation of appetite, and thus feed intake, is entirely appropriate. On return to outdoor pens (PENS), leptin levels were further reduced in both treatment groups even though DMI was close to the PreChallenge mean. Chelikani et al. (2004) observed similar behaviour on refeeding 2 and 3-day fasted heifers and non-lactating cows. This scenario curtails the anorexigenic influence of leptin in the early phase of re-establishing feed intake and initiating compensatory gain. With much increased DMI and weight gain in Late PENS, plasma leptin returned to the PreChallenge mean in both groups.

Adiponectin best discriminated between the two groups. Like leptin, adiponectin is expressed mostly by adipose tissue, but its secretion decreases with increased adipose mass (reviewed Lee and Shao 2014; Khoramipour et al. 2021). Most reports on circulating adiponectin in healthy humans and rodent models indicate no response to fasting or feed restriction; the consensus being that circulating adiponectin levels do not reflect acute reductions in feed intake (Imbeault 2007). In the lactating Holstein, Singh et al. (2014) observed no change in plasma adiponectin concentrations during feed restriction and realimentation. Not surprisingly then, the FRTN group showed no change in plasma adiponectin concentration during feed restriction and, whilst there was a substantial increase during realimentation (Recovery), no relationship of plasma adiponectin concentration with DMI was discerned.

Reports of adiponectin responses during increased heat load in ruminants are limited. A 5-day moderate heat stress mouse model revealed the hyperthermic group exhibited increased circulating adiponectin and adipose expression relative to the thermoneutral feed restricted group (Morera et al. 2012). Contrary to this model, the TC steers in this study showed reduced plasma adiponectin, which returned to normal with cooling and resumption of feeding. Furthermore, across both treatment groups, a strong negative linear relationship between plasma adiponectin with rumen temperature was evident. The only other instance associating adiponectin with core temperature was a study conducted by Wei et al. (2017) whereby adiponectin-null mice were unable to maintain core temperature during short-term cold stress. Moreover, unlike the FRTN group, the TC group displayed a strong and positive linear relationship with DMI. Thus, plasma adiponectin was at its lowest concentrations when rumen temperatures were highest, and DMI at its lowest suggesting other inputs to the regulation of adiponectin secretion than white adipose mass alone. However, in PENS and Late PENS, as live weight and presumably adipose mass increased in both groups, plasma adiponectin concentrations fell and stabilised.

### Synthesis

Having addressed the responses of each hormone independently, this section attempts to integrate the findings to understand the metabolic hormonal milieu of the treatment groups during the Challenge and Recovery periods, and feedlot finishing. Furthermore, the metabolic consequences for these steers are discussed also.

#### Challenge

The nature of the two perturbations imposed in this study, one of reduced feed intake and realimentation, and other of moderate heat load and recovery, were not sufficient to significantly alter plasma TSH and prolactin concentrations. However, a negative linear relationship between plasma TSH and rumen temperature suggests that higher heat load and higher core temperatures than those imposed and observed in this experiment could significantly diminish TSH secretion by the pituitary. The downstream endocrine organs, the thyroid, pancreas and adipose depots, did respond to the perturbations. The two groups were differentiated by insulin, T4, leptin and adiponectin levels, during Challenge and to some extent in Recovery. Moreover, the earlier report on the metabolic responses of these same animals revealed that during Challenge the two treatment groups were differentiated by various energy metabolites, namely plasma concentrations of glutamine, triglycerides (TG) and cholesterol. These were all increased in the FRTN group (Wijffels et al. 2022). On the other hand, NEFA and glucose concentrations were not altered in either group during Challenge. In fact, both treatment groups were euglycaemic through all periods with minor fluctuations.

In Challenge, both plasma T4 and insulin unexpectedly rose in the FRTN group. In an underfed state, these hormones typically fall in plasma. In a stress state, these hormones are known to increase as part of an allostatic response. The atypical increases in levels of insulin and T4 for the FRTN group may be a response to confinement and/or unpredictable chronic handling stress. Meanwhile, both plasma leptin and adiponectin reacted to underfeeding and realimentation as anticipated from numerous other studies. Thus, the metabolic hormone milieu that arose in the FRTN group during Challenge was one of reduced leptin levels, increased insulin and T4 concentrations and unchanged plasma adiponectin. Under conditions of elevated plasma insulin and euglycaemia, as in the FRTN group, insulin promotes release of glutamine into circulation (Meyer et al. 2004), thus contributing to the rise in plasma glutamine. Moreover, elevated levels of circulating thyroid hormones are known to increase the release of glutamine from some skeletal muscles (Parry-Billings et al. 1990). The combined actions of insulin and T4 on skeletal muscle and liver would promote *uptake* of glucose, amino acids and fatty acids, in turn drive gluconeogenesis, glycogen synthesis and protein synthesis. The higher level of insulin would have enhanced lipogenesis in the adipose tissues and liver.

Insulin also promotes cholesterol synthesis by enhancing dephosphorylation of HMG-CoA reductase, activating the enzyme consequently favouring cholesterol synthesis. This would have enabled the rise of plasma cholesterol in the FRTN animals. The higher T4 levels encourages cholesterol synthesis also. Meanwhile, the combination of increased plasma T4 and reduced plasma leptin may have augmented insulin sensitivity and possibly insulin secretion. The fall in plasma leptin enhanced appetite, and diminished its inhibitory action on hepatic glucose production, and reduced lipolysis. The lowering leptin levels promotes many of insulin’s effects on hepatic lipid synthesis and metabolism (reviewed Lago et al. 2009). Overall, the FRTN group during the Challenge and in Recovery favoured uptake of glucose, fatty acids and probably amino acids, to maintain an anabolic state in laying down glycogen, lipid and protein.

The TC group, under moderate heat load, saw unchanged plasma T4 and insulin levels, and these were lower than their FRTN counterparts. The TC steers apparently did not or could not respond to the putative confinement and/or unpredictable chronic handling stress in a comparable manner. An interpretation is that in this case the heat stress metabolic hormone response dominated the allostatic response that occurred in the FRTN group. The most distinctive change in the TC group during Challenge was the fall in adiponectin levels. Consequences of reduced circulating adiponectin are diminution of signalling that promotes skeletal muscle fatty acid oxidation and glucose uptake, and adipose release of fatty acids. However, it lessens the inhibitory influence on hepatic gluconeogenesis, glycogenesis and TG synthesis (reviewed Khoramipour et al. 2021). The lower plasma adiponectin may contribute also to reduced insulin sensitivity in muscle and liver. The plasma leptin level was also reduced but to a lesser extent than the FRTN group. This is likely to have contributed to inappetence in the heat stressed steer, and suppressed glucose uptake by muscle and fat. With no change to plasma insulin and T4 during Challenge, TC group may have tended towards conservation of energy, and reduced energy metabolism and limited the anabolic capacity in what should be a rapidly growing animal.

#### Recovery

Insulin and leptin concentrations remained unchanged in the FRTN group compared to Challenge, but the steers experienced a rise in plasma adiponectin and a further rise in plasma T4. This milieu is likely to reinforce the anabolic activity in the FRTN group during Recovery. The TC group during Recovery showed remarkably similar trajectories for these same hormones but with reduced concentrations in each case compared to the FRTN group. The TC group appear to be transitioning/returning to a more anabolic state during this period with the higher levels of T4 and adiponectin promoting insulin sensitivity and uptake of energy metabolites.

#### Feedlot finishing

PENS and Late PENs: Once the steers had returned to the outdoor feedlot environment and resumed weight gain, the hormone concentrations of the two groups converged. There was no difference in the circulating levels of the two groups. However, with increased age, weight and feed intake, the metabolic hormone profiles did not revert to that of the Pre-Challenge state. Leptin levels initially fell in PENS, encouraging feed intake, and then recovered in Late PENS when DMI and live weight were highest (Wijffels et al. 2022). T4 levels also fell in PENS, then recovered to be slightly higher than the PreChallenge levels. Adiponectin levels fell and stabilised, consistent with gradual increase in fat deposition. The increasing plasma insulin and (log_2_)insulin:glucose ratio, higher leptin and low adiponectin are suggestive of insulin resistance in the feedlot finishing phase.

## Conclusion

The limitations of this study are acknowledged. Ideally, a thermoneutral ad lib fed control group would have been run in parallel with the TC and FRTN groups. The ad lib fed group would have enabled a full assessment of the impact of reduced feed intake on the plasma concentrations of these hormones in rapidly growing grain fed steers. The substantial body of literature was used to fill this gap. Finally, there is the potential confounding factor of a confinement/handling stress which may have been reduced with a longer period of familiarisation in the CCR before commencement of the experiment (although such is limited by animal ethics concerns). These steers are relatively young, having never experienced indoor facilities or underfeeding at any stage; the continuous 18-day confinement and the anxiety over insufficient feed may have induced a stress state. The transient mild hyperthermic response in the FRTN group observed by Sullivan et al. (2022) is further evidence of a stress response in this group.

Overall, this study depicted the homeorhetic responses during Challenge (be it feed restriction or moderate heat load), Recovery and finally, finishing in feedlot environment. Homeorhesis can be thought of as a trajectory to a different but appropriate state to enable adjustment to a new environment. Homeorhetic responses are reversible if the environment reverts to its previous state (Collier et al. 2019). The moderate heat load, typical of a Queensland summer, imposed on these animals was well within the homeorhetic range of these feedlot cattle. We compared the responses over time to two perturbations to physiology and endocrine status that invoked differing homeorhetic mechanisms or differing extents of the same homeorhetic mechanisms. We were able to differentiate the trajectories of the metabolic hormone responses to the two different perturbations during Challenge and to some extent in Recovery. Clearly recovery is not immediate. However, there may have been an overlay of an allostatic response to confinement/handling stress detected in the FRTN group. Interestingly, the TC group appeared to have limited capacity to respond to this potential stressor. The implication is that behavioural responses that provoke increased metabolic activity and consequent endogenous heat production are curtailed even under moderate heat load.

## Supplementary Information

Below is the link to the electronic supplementary material.

Supplementary file1 (PDF 75.2 KB)
