# Supplementary material for: Comparing the responses of grain fed feedlot cattle under moderate heat load and during subsequent recovery with those of feed restricted thermoneutral counterparts: metabolic hormones
Source: Int J Biometeorol. 2023 Apr 11;67(5):897–911. doi: 10.1007/s00484-023-02464-w (PMC10167112; doi:10.1007/s00484-023-02464-w)
Supplement: Supplementary file 1 — Supplementary file1 (PDF 75.2 KB) [file 484_2023_2464_MOESM1_ESM.pdf]

Supplementary tables.

Supplementary Table 1. Plasma hormone assay capture and detection antibodies.

Analyte	Capture Antibody	Supplier	Conc. (μg/mL)	Detection Antibody	Supplier	Conc. (μg/mL)
Prolactin	Chicken anti-bovine prolactin	In-house	0.5	Chicken anti-bovine prolactin Biotin	In-house	3.0
Adiponectin	Rabbit anti-bovine adiponectin	In-house	0.27	Chicken anti-bovine adiponectin	In-house	0.425
Leptin	Chicken anti-bovine leptin	In-house	1.25	Rabbit anti-bovine leptin	In-house	0.6
TSH	Mouse monoclonal anti-TSH	Abcam ab6069	0.5	Mouse monoclonal anti-TSH/HRP	Abcam ab6070	0.7
T4	Mouse monoclonal anti-thyroxine	Santa Cruz SANTSC-57454	0.5	-	-	-

Supplementary Table 2. Assay performance (mean±SEM)

Analyte	LoB	LoD	Intra-assay %CV	Inter-assay %CV
Prolactin (ng/mL)	10.6±0.4	16.3±1.0	2.8±0.2	14.8±1.2
Adiponectin (μg/mL)	2.5±1.3	3.0±1.6	4.06±0.33	11.2±1.3
Leptin (ng/mL)	0.7±0.1	0.9±0.1	2.43±0.20	9.0±1.7
TSH (ng/mL)	0.6±0.0	0.7±0.0	2.56±0.13	5.9±0.27
T4 (nM)	0.2±0.0	0.4±0.0	6.31±0.39	15.0±1.2

LoB: Limit of the Blank; LoD: Limit of Detection.

Supplementary Figure

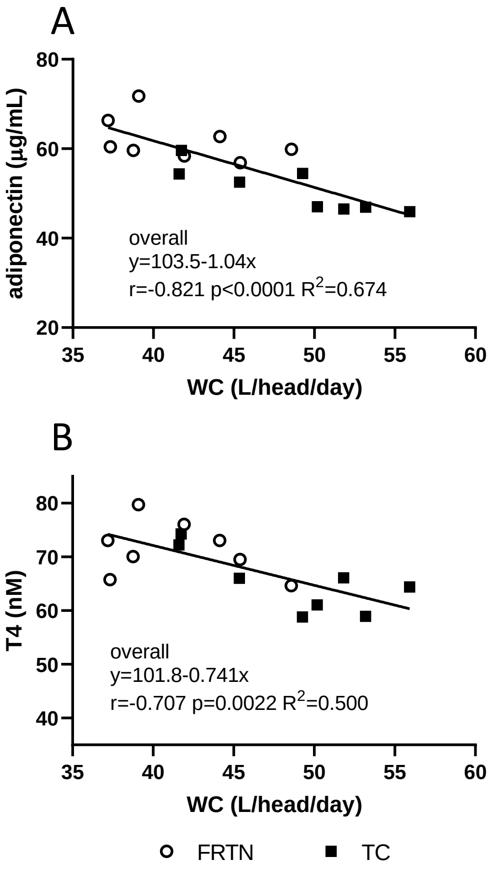

Supplementary Figure 1. Linear relationships between daily mean adiponectin and T4 concentrations and daily mean water consumption (WC) of the thermally challenged (TC) and feed restricted thermoneutral (FRTN) groups during the three periods in the CCR. A. Adiponectin vs WC. B. T4 vs WC. The line-of-best fit and linear equation are given for the data pooled from both treatment groups (overall) or for each treatment group (TC and FRTN) along with the Pearson correlation r, the level of significance and the coefficient of determination, R^2. NS, not significant.
